# Reviewed article: Pinheiro TP, Warmling D, Hofelmann DA, Coelho EBS. Completeness, consistency, and duplicity of self-mutilation reports by adolescents in the Notifiable Diseases Information System: evaluation study, Santa Catarina, 2014-2023. Epidemiol Serv Saude. 2026:35;e20250412

**DOI:** 10.1590/S2237-96222026v35e20250412.b

**Published:** 2025-11-28

**Authors:** Cristine Vieira do Bonfim

**Affiliations:** 1 Fundação Joaquim Nabuco, Recife, PE, Brazil Recife PE Brazil

## First round comments

Estimadas autoras,

O Núcleo Editorial da RESS informa que, após a avaliação, o manuscrito intitulado “Qualidade das notificações de automutilação por adolescentes no Sistema de Informação de Agravos de Notificação em Santa Catarina, 2014-2023” (RESS-2025-0412) foi considerado com potencial para publicação, no entanto reformulações maiores são necessárias. Todos os comentários e sugestões do Núcleo Editorial e dos revisores(as) ad hoc, devem ser considerados e respondidos, item por item, em carta aos editores(as). Deve-se informar a página e as linha onde constam as alterações realizadas. Para as sugestões que forem atendidas, deverá ser realçada a alteração realizada no manuscrito, utilizando-se o amarelo. As sugestões que não forem consideradas deverão ser justificadas com fundamentação científica. A versão reformulada do manuscrito deve considerar as instruções aos autores da RESS e os itens de padronização indicados na revisão técnica. Solicita-se, gentilmente, que as autoras não deixem para enviar o manuscrito no último dia, pois o sistema não é nacional e sempre irá expirar o prazo no dia limite.

Comentários maiores

O manuscrito necessita de uma profunda revisão linguística. Observa-se problemas de redação em todas as seções.

Necessário revisar e adequar o manuscrito às normas da RESS.

Título

Sugere-se a inclusão do desenho do estudo.

Resumo

A leitura do resumo deve possibilitar a compreensão do estudo, sem a necessidade da leitura do trabalho na íntegra. Consiste em uma das seções mais acessadas de um artigo. Por isso, é relevante que seja o mais completa possível, diante do número máximo de palavras, estabelecido pela RESS. Solicita-se, que as autoras revisem com apreço essa seção.

No objetivo é necessário informar que se trata de adolescentes.

Os métodos foram sucintamente descritos. Iniciar com o desenho do estudo. A forma atual parece repetir o objetivo. Quais variáveis foram analisadas? As obrigatórias, as essenciais? Informar ao menos o quantitativo de variáveis analisadas. Nos resultados menciona-se que a completude foi boa e excelente, porém os critérios utilizados para essa classificação não foram apresentados. As técnicas aplicadas na análise dos dados devem ser incluídas. Por exemplo: os resultados indicam um aumento de 32,5 vezes, como foi feita essa análise?

Resultados

O dado sobre aumento, está incompleto, necessário informar o valor do ano inicial e do final da série.

As frequências relativas devem ser acompanhadas das absolutas das quais são derivadas.

A conclusão repetiu os resultados.

Descritores

Sugere-se substituir “Sistemas de Informação em Saúde” por “Sistema de Informação de Agravos de Notificação” e incluir o desenho do estudo.

Introdução

A introdução precisa ser revisada com bastante atenção. A maior parte da seção é sobre automutilação, a qualidade da informação, que é o objetivo do estudo aparece somente no penúltimo parágrafo. A automutiliação precisa ser explicitada e dados epidemiológicos sobre o Brasil e o mundo devem ser incluídos. Observa-se a presença de repetições de informações e a presença de parágrafos soltos. Necessário trabalhar um pouco mais na sequência e conexão entre os parágrafos.

Sugere-se iniciar diretamente com o tema do estudo.

Página, linha 55 - incluir a fonte que fundamenta a definição de automutilação.

Página 2, terceiro parágrafo- Menciona décadas de aumento, entretanto nenhum dado foi apresentado. Sugere-se que as informações sejam expressas com exatidão, onde, qual o período, de quanto foi esse aumento. Trata-se de um parágrafo de frase única.

A seção precisa ser novamente organizada, considerando a sequência cronológica dos eventos. Por exemplo na página 2, o terceiro parágrafo se refere ao ano de 2018, o seguinte 2013, o próximo 2011 e o posterior 2019.

Página 2, linha 56 - evitar afirmativas genéricas “a literatura”. Cita-se a escassez de estudos, para tanto seria necessário a realização de um estudo de revisão sistemática.

Incluir a justificativa para o estudo, explicitando a sua relevância e inovação.

O objetivo relatado no final da introdução deve ter exatamente a mesma redação do apresentado no resumo (copiar e colar).

Métodos

Iniciar a seção com a definição clara e precisa do desenho do estudo.

O item sobre o desenho do estudo, está trazendo também a população. Deve-se observar que existe um item exclusivo para população do estudo.

O período do estudo precisa ser justificado.

Informar a data de acesso aos dados e detalhar didaticamente como os dados foram obtidos. Sugere-se a inclusão de um fluxograma demonstrando a seleção dos registros, com as inclusões e exclusões.

No item sobre as variáveis aparece a “tendencial temporal”, que é análise de dados.

Questiona-se a análise de variáveis de preenchimento obrigatório.

Necessário apresentar definições claras para os critérios analisados na qualidade das notificações. Qual a definição de completude? Qual a fonte?

A seção deve ser organizada de uma forma que possibilite que as definições de completude e consistência fiquem próximas dos critérios adotados para a sua classificação.

Com relação a análise de duplicidade, houve a checagem automatizada e manual. Nessa última é importante citar as métricas aplicadas na validação (sensibilidade, especificidade e o tratamento de homônimos).

A análise de dados precisa ser mais detalhada. Uma questão essencial, é que a regressão de Prais-Winsten foi utilizada para avaliar apenas 10 pontos. Esse foi um dos principais itens apontados na avaliação do manuscrito. Tal fato reduz o poder estatístico do estudo.

Resultados

A seção deve ser redigida com frases curtas e diretas. Tem frases extensas, que comprometem a compreensão do resultado. Observe-se que o segundo parágrafo é constituído por uma única frase.

A Figura 2 é uma distribuição do número de notificação por ano. Deve-se aplicar também a análise de tendência.

Faltam dados numéricos na descrição dos resultados da completude.

Todos os resultados devem fazer menção as tabelas.

Sugere-se a inclusão da análise de tendência para a consistência. Uma vez que se trata de um estudo que aplicou a análise de tendência, por que aplicar para a completude e não para a consistência?

Os resultados não devem ser interpretados. Página 5, linha 17 o termo maioria é interpretativo.

Os títulos das tabelas necessitam ser adequados às normas da RESS.

Sugere-se que os parâmetros utilizados para a classificação da completude e da consistência sejam expostos nas legendas das tabelas. Com isso, não é necessário retornar a seção de métodos para lembrar.

Discussão

A seção inicia com um resultado que não foi descrito anteriormente.

As autoras afirmam que a qualidade do banco foi considerada elevada nos três critérios de qualidade da informação. Parecem desconsiderar que foram identificadas 1.403 notificações de automutilação, após a exclusão de mais de 30% dos registros por problemas no preenchimento do campo referente ao tipo de violência.

Seria interessante tentar explicar a ausência de duplicidade em um banco do Sinan.

A seção de discussão não deve conter dados numéricos de resultados.

O estudo fez uma distribuição do número de notificações por ano. Precisa aplicar o modelo de regressão para analisar a tendência das notificações.

A discussão deve comparar os resultados com os de outros estudos e buscar explicações para os achados. Observa-se que os resultados são apenas comparados, sem o devido aprofundamento na busca de explicações.

Vários parágrafos não fazem a conexão entre os resultados e a literatura. Somente tem-se a literatura.

Outro ponto é que os resultados estão sendo discutidos com pesquisas realizadas em Santa Catarina. Importante comparar com outros estados e com estudos internacionais.

A discussão deve seguir a mesma sequência dos resultados. Muitas das variáveis analisadas na completude não foram discutidas.

Embora reconheçam a limitação referente à falta ou preenchimento inadequado do campo aberto correspondente ao tipo de violência autoprovocada e a baixa completitude em variáveis fundamentais (como “motivação da violência”), as autoras classificam a qualidade das notificações é “boa” ou “excelente” na maioria dos critérios. Pondere-se que a conclusão poderia ser mais crítica. Essa interpretação pode ser classificada como excessivamente otimista e não compatível com os próprios achados, além de desconsiderar o viés de exclusão. Essa dissonância pode comprometer a credibilidade do estudo e gerar interpretações equivocadas por parte de gestores e formuladores de políticas públicas.

Falta uma conclusão para o artigo que responda diretamente ao objetivo do estudo.

Referências

As referências necessitam de atualização.

Comentários menores

Revisar a introdução tem várias frases iniciando da mesma forma.

Página 2, linha 8 - incluir a referência.

Página 2, linhas 9, 10 e 13 - repetição de termos próximos (maneira).

Página 2, linha 9 - redundância (maneira e forma).

Página 2, linha 14 - cita estudos, porém não incluiu as referências. Menciona vários países, sem especificar quais. Acrescente-se que considera recente um artigo publicado em 2020 e isso depende do padrão utilizado. Para alguns recente seria nos últimos três anos, para evitar essas questões, sugere-se que os adjetivos sejam evitados na redação científica.

Evitar expressões desnecessária - é importante ressaltar; nesse contexto, o presente estudo.

Recommendation: Major revision

## Second round comments

Estimadas autoras,

O Núcleo Editorial da revista Epidemiologia e Serviços de Saúde (RESS), após a análise da reformulação do manuscrito intitulado “Avaliação da completitude, consistência e duplicidade das notificações de automutilação por adolescentes no Sistema de Informação de Agravos de Notificação em Santa Catarina, 2014 a 2023” (RESS-2025-0412.R1), sugere, ainda, que sejam realizadas adequações de acordo com o parecer abaixo.

Todos os comentários e sugestões devem ser considerados e respondidos, item por item, na carta aos editores, especificando página e parágrafos nas quais as alterações foram realizadas. Para as sugestões que forem atendidas, deverá ser realçada a alteração r utilizando-se o amarelo. As sugestões que não forem consideradas deverão ser justificadas com fundamentação científica.

Resumo

Substituir 7 por sete.

A frase inicial da seção de resultados, está repetindo o período de referência do estudo.

A seção de resultados carece de dados numéricos. A forma atual parece um texto de discussão.

Substituir doze por 12. Somente números inferiores a 10 devem ser escritos por extenso.

Introdução

A primeira frase do segundo parágrafo necessita de referência. Observa-se a ausência de um sujeito nessa frase. Quem representa? Acrescente-se a necessidade de apresentar uma prevalência estimada para o mundo. São apresentados alguns estudos, que tratam de prevalências para os locais pesquisados. Importante acrescentar nesse parágrafo que a prevalência de automutilação é variável entre os países e envolve diversos fatores. Poderiam, inclusive, explorar alguns.

Terceiro parágrafo repetição de termos próximos (estudo). Neste mesmo parágrafo observa-se a citação de alguns estudos desenvolvidos no Brasil. Importante fazer uma conexão entre eles. Lembrar que as prevalências são variáveis.

Quarto parágrafo, onde foi observado esse aumento? Apresentar os dados que demonstram o aumento e em qual período. Incluir a referência que fundamenta a afirmativa.

Último parágrafo da seção, repetição excessiva do vocábulo “estudo”.

Métodos

Substituir o termo metodologia por métodos

A primeira frase do item delineamento está repetindo o objetivo, pode ser removida.

Incluir uma referência que fundamente a classificação do estudo como de avaliação.

No segundo parágrafo incluir uma referência sobre a mudança da ficha. Ainda sobre a mudança da ficha, esse seria o motivo para não ampliação da série? Considerando que o número de pontos, foi considerado uma limitação relevante e fundamentou um parecer que indicou a rejeição. Seria interessante trazer a questão da mudança da ficha para a discussão da limitação, que se refere ao número de pontos temporais.

Justificar também o ano final da análise, muito provavelmente eram os dados fechados no sistema no momento de realização do estudo. Isso precisa ser especificado.

No item participantes, deve-se incluir a definição de automutilação adotada e como foi obtida.

No item variáveis, por que as obrigatórias foram analisadas? Considerando que sem o seu preenchimento não é possível avançar na digitação da ficha. E elas aparecem nos resultados com 100%.

Substituir raça/cor por raça/cor da pele. Revisar em todo o texto.

Incluir uma referência para a definição de completude.

Incluir o número da referência de Romero e Cunha.

Citar a fonte utilizada para a classificação da violência.

Qual o critério utilizado para a seleção das variáveis analisadas na consistência? Justificar a seleção dessas variáveis.

No item fonte de dados, tem informações que se referem à população do estudo. Sugere-se deslocar a informação para esse item.

Última frase do item fonte de dados necessita de referências.

O controle de viés informa que foram “analisadas as notificações de automutilação,

por adolescentes, em Santa Catarina de 2014 a 2023 com exclusão, manualmente, das

notificações em que o campo “outros” estava vazio ou preenchido com informações

insuficientes para a identificação clara do tipo de violência”. Essa análise deveria ser apresentada em uma tabela suplementar.

O tamanho do estudo parece população do estudo. Sugere-se retirar.

A figura 1 é método ou resultado? Caso seja a primeira opção não seria a forma de seleção da população do estudo?

Resultados

A análise de tendência do número de notificações não é objetivo desse estudo. Sugere-se removê-la.

Último parágrafo da seção a análise da tendência da consistência necessita carece de dados numéricos.

Figura 2 - A figura trata da tendência temporal das notificações de automutilação, embora se relacione com o tema do estudo, não é este o objeto aqui. Sugere-se retirar essa tabela e se concentrar na análise dos critérios de qualidade.

Discussão

Revisar a seção e retirar todos os dados numéricos de resultados.

Reforça-se que a análise de tendências da notificação não faz parte do objetivo desse estudo, que se concentra na análise da qualidade da informação.

Retirar a explicação da sigla Sinan, já foi feita anteriormente.

Segundo parágrafo da página 6, menciona-se estudos, mas não cita. Faltam as referências dos estudos discutidos.

Questiona-se novamente qual a finalidade da análise da completude das variáveis obrigatórias. A explicação sobre variável essencial e obrigatória deve ser deslocada para a seção de métodos.

A discussão das limitações traz a questão da área de estudo, não parece ser uma limitação, foi escolha das autoras.

Uma importante limitação do estudo é o número de pontos temporais. Essa limitação foi apontada pelo revisor, sendo inclusive a fundamentação para a indicação de rejeição. No entanto, isso não foi respondido. Questiona-se por que não houve a ampliação do número de pontos temporais? A análise de Prais -Winsten é relativamente simples e rápida para ser realizada.

A perda de 30% precisa ser discutida fortemente no manuscrito. Além disso, a análise descrita na seção de método deve ser apresentada como material suplementar.

Sobre a afirmativa de pesquisas escassas é necessário a realização de um estudo de revisão sistemática que fundamente e isso não ocorreu. Solicita-se, gentilmente, que essa afirmativa seja suprimida.

Último parágrafo não é limitação do estudo, além de não fazer uma conexão com os resultados do estudo. Parece revisão de literatura.

Falta uma conclusão para o estudo. Pondera-se que que recomendações (sugestões) podem ser feitas no parágrafo anterior a conclusão. No entanto, a conclusão deve ser apresentada no último parágrafo respondendo diretamente ao objetivo.

Revisar a lista de referências em relação as normas técnicas da RESS. Há problemas de formatação e referências incompletas.

O texto necessita de revisão linguística, observa-se a presença de frases extensas, que dificultam a compreensão, além de muitas repetições de termos. Por favor, revisem com atenção, em alguns trechos o entendimento da argumentação fica comprometido.

Recommendation: Minor revision

## Third round comments

Estimadas autoras,

O Núcleo Editorial da revista Epidemiologia e Serviços de Saúde (RESS), após a análise da reformulação do manuscrito intitulado “Avaliação da completitude, consistência e duplicidade das notificações de automutilação por adolescentes no Sistema de Informação de Agravos de Notificação em Santa Catarina, 2014 a 2023”, sugere, ainda, que sejam realizadas adequações de acordo com o documento anexo.

Todos os comentários e sugestões devem ser considerados e respondidos, item por item, na carta para os editores. Para as sugestões que forem atendidas, deverá ser realçada a alteração realizada no manuscrito, utilizando-se o amarelo. As sugestões que não forem consideradas deverão ser justificadas com fundamentação científica.

Cordialmente,

Núcleo Editorial

Epidemiologia e Serviços de Saúde: revista do Sistema Único de Saúde do Brasil

Site: www.scielo.br/ress

Comentários

Página 2, linha 49 - a afirmativa sobre escassez de literatura somente deve ser realizada, quando fundamentada por um artigo de revisão sistemático. Tal fato, não se aplica aqui. Sugere-se que essa afirmativa seja removida.

Página 3, primeiro parágrafo - remover a expressão “o presente artigo”. Juntar a frase que contêm o objetivo com o parágrafo anterior.

No objetivo retirar a sigla Sinan.

Página 6, primeiro parágrafo - parece trazer uma discussão de aumento da tendência referente ao resultado que foi retirado. Sugere-se suprimir esse parágrafo.

Página 7, linha 15 - o termo “corroborou” é bastante forte, tem o sentido de comprovação. Sugere-se a sua substituição.

Página 7, parágrafo que inicia na linha 51 - sugere-se a sua remoção. Não houve um estudo de revisão sistemática que fundamente tal afirmativa.

Página 8, a última frase da conclusão deve ser deslocada para o final do parágrafo que aborda as limitações do estudo.

Incluir o material suplementar solicitado na versão anterior.

Recommendation: Minor revision

